# Association between dietary branched-chain amino acid intake and anaemia risk in children and adolescents: a matched case-control study

**DOI:** 10.3389/fnut.2026.1911612

**Published:** 2026-09-28

**Authors:** Xuan Liu, Shuyi Wu, Yulin Jiang, Changqing Liu, Yiya Liu, Meina Tian, Qianrang Zhu, Qing Yue, Zhenchuang Tang, Lianlong Yu

**Affiliations:** 1 School of Public Health, Shandong Second Medical University, Weifang, China; 2 Hebei Center for Disease Control and Prevention, Shijiazhuang, China; 3 Guizhou Center for Disease Control and Prevention, Guiyang, China; 4 Jiangsu Provincial Center for Disease Control and Prevention, Nanjing, China; 5 National Center for Women and Children's Health, National Health Commission, Beijing, China; 6 Institute of Food and Nutrition Development, Ministry of Agriculture and Rural Affairs, Beijing, China; 7 Institute of Public Health Surveillance and Evaluation, Shandong Center for Disease Control and Prevention, Jinan, China

**Keywords:** anaemia, branched-chain amino acids, case-control study, children and adolescents, nutritional epidemiology, propensity score matching

## Abstract

Anaemia in children and adolescents is multifactorial, and evidence on dietary amino-acid intake in relation to anaemia remains limited. This study examined the association between dietary branched-chain amino acid (BCAA) intake and haemoglobin (Hb)-defined anaemia among Chinese children and adolescents using data from the 2016–2019 China Children and Lactating Women Nutrition and Health Survey. Among 11,134 eligible participants aged 6–17 years, quantile g-computation and the Boruta algorithm were first used to screen 20 dietary amino acids. A 1:4 propensity score-matched sample was then constructed, including 455 participants with anaemia and 1,819 with normal Hb levels. Dietary BCAA intake and its components, isoleucine (Ile), leucine (Leu) and valine (Val), were energy-adjusted using the residual method. Machine learning methods were used exploratorily for feature ranking, and conditional logistic regression was used to estimate odds ratios (ORs). After matching, total dietary protein intake was comparable between groups, whereas intakes of total BCAA, Ile, Leu, and Val were significantly lower in the anaemia group. In fully adjusted models, higher intakes of total BCAA, Ile, Leu, and Val were associated with lower odds of Hb-defined anaemia, with ORs of 0.95 (95% CI: 0.92–0.99), 0.82 (95% CI: 0.70–0.95), 0.91 (95% CI: 0.85–0.98) and 0.86 (95% CI: 0.78–0.95), respectively. Supplementary biomarker analyses showed lower ferritin and higher soluble transferrin receptor concentrations in the anaemia group, whereas high-sensitivity C-reactive protein did not differ between groups. These findings suggest that BCAA-related dietary characteristics may provide useful nutritional clues for anaemia-related assessment, although the association should not be interpreted as evidence of a BCAA-specific protective effect.

## 1 Introduction

Anaemia remains a major public health problem worldwide, particularly among children and adolescents, and it has not yet been fully eliminated in Chinese paediatric populations. Identifying modifiable dietary factors is therefore important for improving nutritional assessment and health management of anaemia in children and adolescents (1, 2). Branched-chain amino acids (BCAAs), including isoleucine (Ile), leucine (Leu), and valine (Val), are essential amino acids and key substrates for protein synthesis, energy metabolism, and metabolic regulation (3, 4). Studies have shown that abnormal amino acid metabolism is closely related to the occurrence and development of various diseases. BCAAs may also be associated with iron metabolism, haematopoiesis, and erythropoiesis, suggesting a potential role in the development of anaemia (5, 6).

However, population-based evidence regarding the relationship between dietary BCAA intake and anaemia in children and adolescents remains limited. Previous studies have focused mainly on the associations of dietary patterns or iron deficiency with anaemia, whereas studies directly exploring anaemia risk in relation to individual amino acids, particularly dietary BCAA intake, remain scarce (7–9). Meanwhile, some studies in children have begun to examine the associations between amino acid intake and outcomes such as inflammation using machine learning methods (10), but there is still a lack of research that uses anaemia as an outcome and combines traditional epidemiology with machine learning models for comprehensive analysis.

Accordingly, we used data from the China Children and Lactating Women Nutrition and Health Survey to evaluate the associations between energy-adjusted total dietary BCAA intake and its components (Ile, Leu, and Val) and anaemia risk in children and adolescents. The aim was to provide dietary amino-acid-level evidence for nutritional assessment related to childhood anaemia and public health nutrition research.

## 2 Materials and methods

### 2.1 Study design and participants

This study used data from the China Children and Lactating Women Nutrition and Health Survey (CCLWNHS; 2016–2019). The survey divided 31 provinces, autonomous regions, and municipalities in China into large cities, small and medium-sized cities, general rural counties, and poor rural counties, and selected 150 surveillance sites according to population distribution, comprising 26 large cities, 45 small and medium-sized cities, 46 general rural counties, and 33 poor rural counties. This study selected data from Shandong, Hebei, Jiangsu, and Guizhou provinces. All participants underwent face-to-face interviews, physical examinations, and provided biological samples. All children and adolescents aged 6–17 years had 6 mL of fasting venous blood collected (2 mL anticoagulated blood and 4 mL non-anticoagulated blood). Whole-blood haemoglobin (Hb) was measured on site by laboratory personnel at the surveillance sites using the HemoCue method. Standardised equipment and procedures were used throughout data collection. Ferritin, soluble transferrin receptor (sTfR), high-sensitivity C-reactive protein (hsCRP), albumin, serum total protein, zinc and vitamin A were also measured and used in supplementary biomarker analyses.

All participants and their guardians provided written informed consent. The study protocol was approved by the Ethics Review Committee of the National Institute for Nutrition and Health (NINH), Chinese Center for Disease Control and Prevention (approval no. 201614). Dietary information was collected using a food frequency questionnaire (FFQ) with established reliability and validity to assess habitual dietary intake during the preceding month. Nutrient and amino acid intakes were estimated using the Chinese Food Composition Table, Standard Edition (6th edition) (11). The questionnaire was designed by a team of experts from the NINH and the Chinese Center for Disease Control and Prevention and is a standardised questionnaire that has been validated and widely used in large-scale dietary surveys of Chinese children.

### 2.2 Procedures

This study was based on CCLWNHS data collected between 2016 and 2019 and initially included 23,301 children and lactating women. Database retrieval and screening were completed between August and October 2025. According to the exclusion criteria, 11,440 individuals aged above 17 years or below 6 years were excluded, as were 727 individuals with missing Hb data. After these exclusions, 11,134 participants aged 6–17 years were retained for analysis. Anaemia was defined according to the 2024 World Health Organization Hb cut-offs as follows: Hb < 115 g/L for children aged 6–11 years, Hb < 120 g/L for those aged 12–14 years, Hb < 120 g/L for girls aged 15–17 years, and Hb < 130 g/L for boys aged 15–17 years (12). Based on the above criteria, the study subjects were divided into an anaemia group and a normal Hb group. The primary outcome was Hb-defined anaemia; ferritin, sTfR and hsCRP were used only to support supplementary interpretation of iron and inflammatory status.

In the 11,134 eligible participants, preliminary screening and validation were conducted for 20 dietary amino acids related to anaemia. Quantile g-computation (qgcomp) was used with continuous Hb level as the dependent variable to assess the direction of association between dietary amino acids and Hb. In parallel, the Boruta algorithm was applied with binary anaemia status as the dependent variable to identify amino acids that were important for anaemia risk; the number of Boruta iterations was set at 100. Integrating the qgcomp and Boruta results, Ile, Leu, and Val were identified as important amino acids associated with anaemia. Total BCAA intake and its components Ile, Leu, and Val were therefore selected as the key exposure variables for subsequent analyses.

To control for the effect of total energy intake on dietary BCAA intake, the residual method (13, 14) was used to adjust BCAA, Ile, Leu, and Val, and the corrected values were used in subsequent analyses. On the basis of the existing literature and variable availability, age, sex, BMI, physical activity, and energy intake were selected as the main confounders. Propensity score matching (PSM) was then used to balance confounding between groups. Nearest-neighbour matching with a calliper of 0.25 was performed at a ratio of 1:4, and a standardised mean difference (SMD) < 0.1 after matching was used as the reference criterion for covariate balance. These covariates were additionally retained in the fully adjusted conditional logistic regression model to account for any residual imbalance and to improve the precision of the association estimates after matching.

In the sample following PSM, there were 1,819 participants in the normal group and 455 participants in the anaemia group. Four machine learning models—Extreme Gradient Boosting (XGBoost), Light Gradient Boosting Machine (LightGBM), Categorical Boosting (CatBoost), and naive Bayes (NB)—were then applied. The performance of each model was evaluated using the area under the curve (AUC) of the receiver operating characteristic (ROC). The Shapley additive explanations (SHAP) algorithm was used to assess the relative feature importance of the best-performing model. Conditional logistic regression was used to analyse the association between total BCAA intake and its components and the odds of anaemia. Because total BCAA is the sum of Ile, Leu, and Val, these exposure variables were entered separately into different regression models to avoid multicollinearity. In contrast, the LightGBM model used for SHAP analysis included the exposure variables and covariates simultaneously to explore their relative contributions to model prediction; therefore, SHAP values were interpreted as relative feature importance within the joint model rather than as independent effects. Further sensitivity tests were conducted using subgroup analysis, interaction analysis, and restricted cubic spline (RCS) analysis.

Subgroups were defined by age (6–10, 11–14, and 15–17 years), sex (male and female), BMI (underweight, normal weight, overweight, and obese), physical activity (< 60 min/day and ≥60 min/day), and energy intake (low, medium, and high tertiles). BMI categories were defined according to age-specific Chinese standards for children and adolescents. Interactions were assessed using likelihood ratio tests. In the RCS analysis, total BCAA, Leu, and Val were modelled with four knots at the 5th, 35th, 65th, and 95th percentiles, whereas Ile was modelled with three knots at the 10th, 50th, and 90th percentiles. The number of knots was chosen using a combination of the Akaike information criterion (AIC) and the Bayesian information criterion (BIC), with the median used as the reference value.

### 2.3 Statistical and machine learning methods

Propensity score matching (PSM) estimates the probability of assignment to a given exposure or comparison group conditional on observed covariates. By compressing multidimensional covariate information into a single score and matching on that score, PSM reduces confounding bias in observational studies and improves comparability between groups (15).

qgcomp is a statistical approach for analysing correlated exposure mixtures. It estimates the overall effect and direction of multiple related exposures by discretising continuous exposures into quantiles and entering them into the same model, while allowing individual exposures to contribute with different directions and magnitudes (16).

Boruta is an all-relevant feature selection method based on random-forest importance metrics. It creates randomly permuted shadow features and repeatedly compares the importance of the real variables with that of the shadow variables, thereby identifying features that are genuinely associated with the outcome (17, 18).

XGBoost is an ensemble learning algorithm based on gradient boosting trees that iteratively fits the residuals from previous trees in order to optimise predictive performance. By incorporating regularisation, parallel computation, and missing value handling, it offers both high predictive accuracy and strong computational efficiency (19, 20).

LightGBM is also a gradient boosting decision tree (GBDT) algorithm, characterised by its fast training speed and low memory consumption in large-sample and high-dimensional data. This method improves the learning ability of complex nonlinear patterns by optimising the tree growth strategy and splitting method, while maintaining good generalisability performance (21, 22).

CatBoost is a gradient boosting algorithm specifically optimised for handling categorical variables. Its core improvement lies in reducing target leakage and prediction bias through ordered boosting and ordered target statistics, thereby improving model stability (23, 24).

Naive Bayes (NB) is a class of probabilistic classification models based on Bayes' theorem. Its basic assumption is that the predictor variables are conditionally independent of each other given a class. Although this independence assumption is usually difficult to fully satisfy in real-world data, the model is simple in structure, fast in training, and has good adaptability to high-dimensional data, so it is still widely used in classification tasks (25, 26).

Restricted cubic splines (RCS) are a statistical method for fitting the nonlinear relationship between continuous variables and outcomes. The principle is to construct a smooth curve by using piecewise cubic polynomials and apply linear constraints at both ends of the data range to avoid overfitting (27, 28).

Machine learning analyses were used as exploratory feature-ranking tools rather than to develop a clinically validated prediction model. Owing to the lack of an external validation dataset, AUC values were interpreted descriptively and were not used as evidence of clinical predictive performance. Machine learning was used primarily to identify important amino-acid-related features, after which conditional logistic regression was used to estimate odds ratios and sensitivity analyses were performed.

### 2.4 Statistical analysis

Categorical variables are presented as counts and percentages, and comparisons between groups were performed using the χ^2^ test. Continuous variables were assessed for distribution and are presented as medians with interquartile ranges [*M*(*Q*_1_, *Q*_3_)], and comparisons between groups were performed using the Mann–Whitney *U* test. The qgcomp model and Boruta algorithm were used for preliminary screening and validation of dietary amino acids associated with anaemia. Covariate balance before and after PSM was evaluated using SMD. After energy residual correction of total dietary BCAA intake and its components Ile, Leu and Val, XGBoost, LightGBM, CatBoost and naive Bayes were applied exploratorily for feature ranking. ROC curves and AUCs were used descriptively, and SHAP values were used to summarise relative feature importance in the LightGBM model. Conditional logistic regression analysis was used to analyse the associations between total BCAA, Ile, Leu, and Val and the risk of anaemia, calculating the ORs and 95% confidence intervals (CIs). Subgroup analysis, interaction analysis, and RCS analysis were used to assess the robustness of the associations. All statistical analyses were performed using R (version 4.5.3; R Foundation for Statistical Computing, Vienna, Austria), and a two-sided *P* value < 0.05 was considered statistically significant.

## 3 Results

### 3.1 Baseline characteristics of the study population

This study screened 11,134 children and adolescents aged 6–17 years using inclusion and exclusion criteria (Figure 1A). The qgcomp model and Boruta algorithm were used for preliminary screening and validation of 20 dietary amino acids. The qgcomp results showed that Ile, Leu, and Val were positively associated with Hb levels (Figure 2A), whereas Boruta showed that Ile, Leu, and Val were all classified as confirmed features for anaemia risk (Figure 2B), suggesting they are important amino acids associated with anaemia.

**Figure 1:**
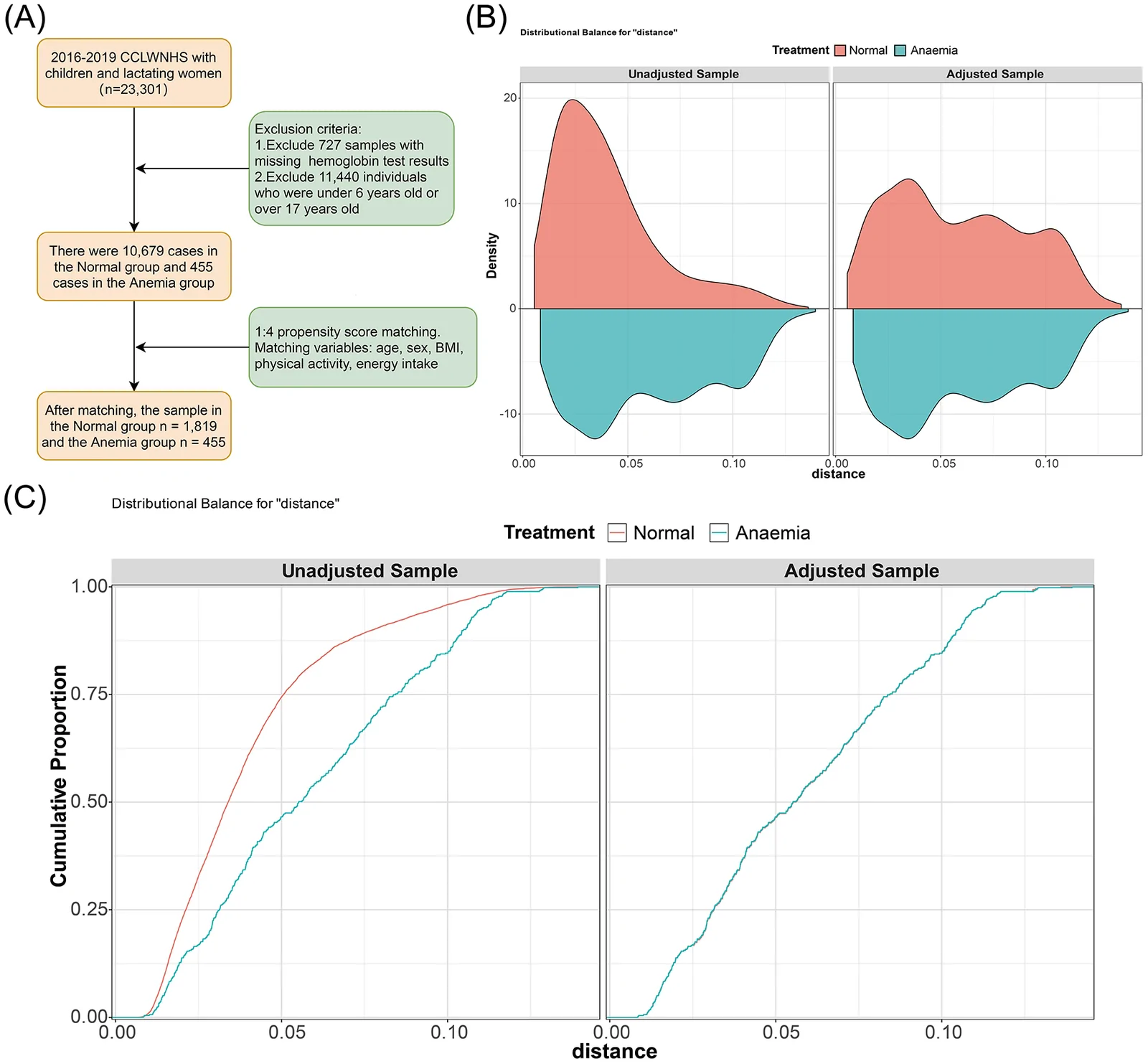
Study flowchart **(A)** and schematic diagrams of propensity score matching **(B,C)**.

**Figure 2:**
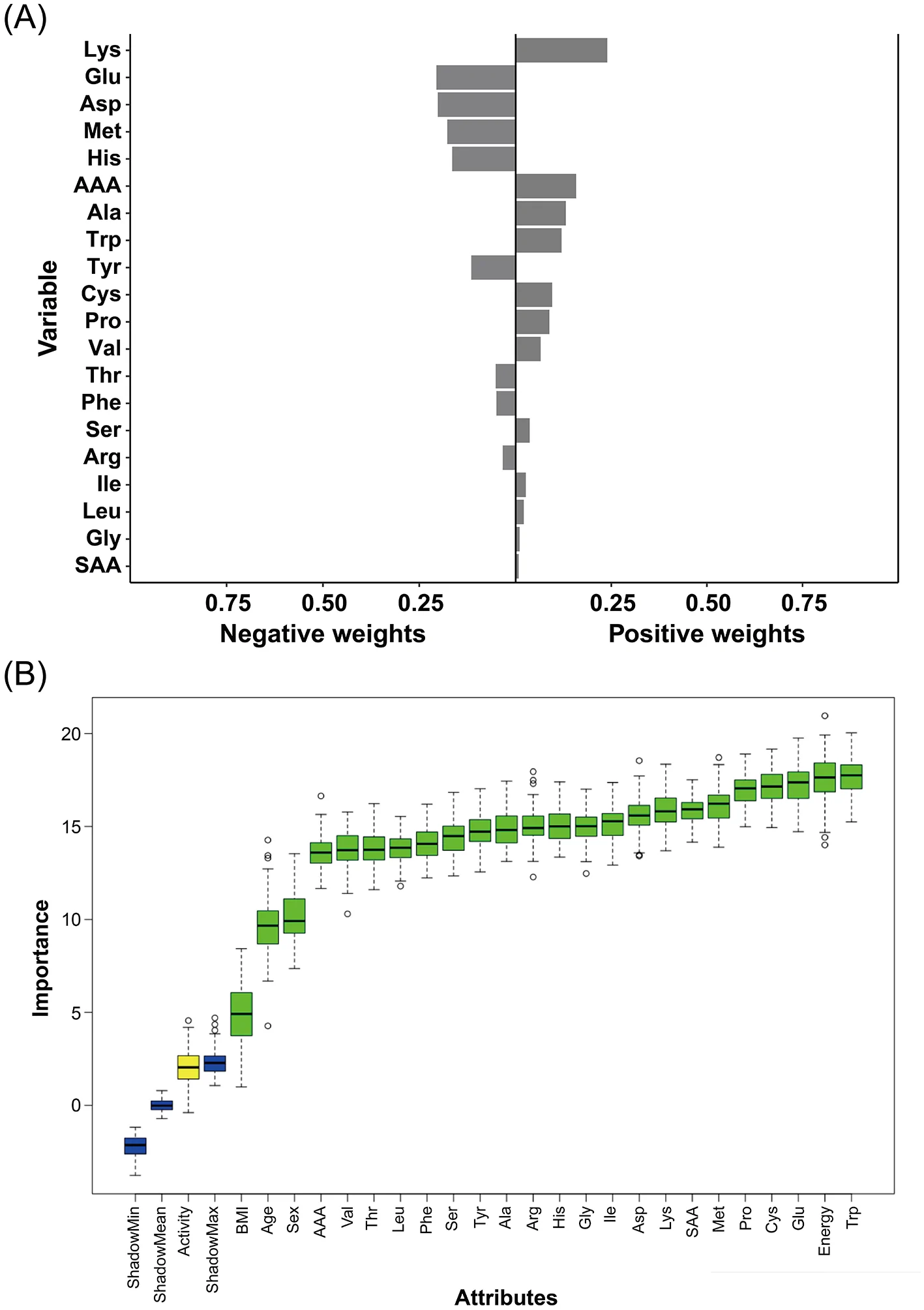
Screening and validation of dietary amino acids associated with anaemia using qgcomp and the Boruta algorithm. **(A)** Contribution weights of 20 dietary amino acids to haemoglobin (Hb) levels. Positive weights indicate positive associations with Hb levels, whereas negative weights indicate negative associations; **(B)** Dietary amino acid features associated with anaemia risk identified by the Boruta algorithm. Green indicates confirmed features; yellow, tentative features; red, rejected features; and blue, shadow features (random reference variables).

Based on the above results, total BCAA and its components Ile, Leu, and Val were subsequently included as key exposure variables in the post-matched analysis. A 1:4 PSM was used to balance confounding factors (Figures 1B, C), ultimately including 2,274 participants, of whom 455 (20%) were in the anaemia group and 1,819 (80%) were in the normal Hb level group. Before matching, there were significant differences between the anaemia group and the normal group in age, sex, BMI, and physical activity (*P* < 0.05). After matching, the distribution of confounding factors between the two groups was balanced, and none were statistically significant (*P* > 0.05), and the SMD decreased significantly, indicating good matching performance (Table 1).

**Table 1:** Comparison of baseline characteristics of the study population before and after propensity score matching.

Variables	Before PSM						After PSM					
	**Total (*****n*** = **11,134)**	**Normal (*****n*** = **10,679)**	**Anaemia (*****n*** = **455)**	**Statistic**	***P***	**SMD**	**Total (*****n*** = **2,274)**	**Normal (*****n*** = **1,819)**	**Anaemia (*****n*** = **455)**	**Statistic**	***P***	**SMD**
Age (years), *M* (*Q*_1_, *Q*_3_)	11.00 (8.00, 13.00)	11.00 (8.00, 13.00)	13.00 (9.00, 16.00)	*Z* = −8.565^b^	**< 0.001**	0.396	13.00 (9.00, 15.00)	13.00 (10.00, 15.00)	13.00 (9.00, 16.00)	*Z* = −0.453^b^	0.651	0.008
Sex, *n* (%)				χ^2^ = 77.642^a^	**< 0.001**					χ^2^ = 0.024^a^	0.876	
Male	5,482 (49.24)	5,350 (50.10)	132 (29.01)			−0.465	653 (28.72)	521 (28.64)	132 (29.01)			0.008
Female	5,652 (50.76)	5,329 (49.90)	323 (70.99)			0.465	1,621 (71.28)	1,298 (71.36)	323 (70.99)			−0.008
BMI (kg/m^2^), *M* (*Q*_1_, *Q*_3_)	17.99 (15.92, 20.83)	17.96 (15.89, 20.78)	19.06 (16.45, 21.37)	*Z* = −4.193^b^	**< 0.001**	0.163	18.87 (16.56, 21.47)	18.84 (16.58, 21.55)	19.06 (16.45, 21.37)	*Z* = −0.072^b^	0.943	0.011
Physical activity (min/d), *M* (*Q*_1_, *Q*_3_)	30.00 (15.00, 60.00)	30.00 (15.00, 60.00)	30.00 (0.00, 60.00)	*Z* = −3.521^b^	**< 0.001**	−0.184	30.00 (10.00, 60.00)	30.00 (10.00, 60.00)	30.00 (0.00, 60.00)	*Z* = −0.281^b^	0.779	0.005
Energy intake (kcal/d), *M* (*Q*_1_, *Q*_3_)	1,711.82 (1,245.46, 2,294.87)	1,712.47 (1,246.23, 2,293.88)	1,679.90 (1,223.81, 2,298.80)	*Z* = −0.019^b^	0.985	−0.010	1,712.98 (1,267.01, 2,273.61)	1,715.85 (1,275.78, 2,263.44)	1,679.90 (1,223.81, 2,298.80)	*Z* = −0.191^b^	0.848	−0.010

a χ^2^ test (*P* < 0.05).

b Mann–Whitney *U* test (*P* < 0.05).

*M*, median; Q_1_: 1st quartile; Q_3_: 3rd quartile; SMD, standardised mean difference. Bold values indicate statistically significant results (*P* < 0.05).

### 3.2 Association between dietary branched-chain amino acids and anaemia risk

The baseline characteristics of the matched population are shown in Table 2. After PSM, dietary protein intake did not differ significantly between the normal haemoglobin group and the anaemia group [96.97 (69.56, 129.42) vs. 92.68 (63.68, 131.28) g/d, *P* = 0.156]. In contrast, median intakes of total BCAA, Ile, Leu and Val were significantly lower in the anaemia group (*P* < 0.01). These findings suggest that the observed differences in BCAA intake were not simply accompanied by a statistically significant difference in total protein intake. Violin plots of Hb levels by quartiles of these amino acids showed differences across quartile groups for total BCAA, Leu and Val (*P* < 0.05), although pairwise comparisons did not show a strictly monotonic pattern (Figure 3).

**Table 2:** Baseline characteristics of the study population after propensity score matching.

**Variables**	Total (*n* = 2,274)	Normal (*n* = 1,819)	Anaemia (*n* = 455)	Statistic	*P*
Age (years), *M* (Q_1_, Q_3_)	13.00 (9.00, 15.00)	13.00 (10.00, 15.00)	13.00 (9.00, 16.00)	*Z* = −0.45^b^	0.651
Sex, *n* (%)				*χ^2^* = 0.02^a^	0.876
Male	653 (28.72)	521 (28.64)	132 (29.01)		
Female	1,621 (71.28)	1,298 (71.36)	323 (70.99)		
BMI (kg/m^2^), *M* (*Q*_1_, *Q*_3_)	18.87 (16.56, 21.47)	18.84 (16.58, 21.55)	19.06 (16.45, 21.37)	*Z* = −0.07^b^	0.943
Physical activity (min/d), *M* (*Q*_1_, *Q*_3_)	30.00 (10.00, 60.00)	30.00 (10.00, 60.00)	30.00 (0.00, 60.00)	*Z* = −0.28^b^	0.779
Energy intake (kcal/d), *M* (*Q*_1_, *Q*_3_)	1,712.98 (1,267.01, 2,273.61)	1,715.85 (1,275.78, 2,263.44)	1,679.90 (1,223.81, 2,298.80)	*Z* = −0.19^b^	0.848
Protein intake (g/d), *M* (*Q*_1_, *Q*_3_)	96.31 (68.59, 129.58)	96.97 (69.56, 129.42)	92.68 (63.68, 131.28)	*Z* = −1.42^b^	0.156
BCAA (g/d), *M* (*Q*_1_, *Q*_3_)	16.27 (13.93, 18.88)	16.46 (14.03, 19.05)	15.72 (13.56, 18.02)	*Z* = −3.11^b^	**0.002**
Ile (g/d), *M* (*Q*_1_, *Q*_3_)	3.74 (3.28, 4.27)	3.76 (3.30, 4.31)	3.66 (3.21, 4.11)	*Z* = −2.88^b^	**0.004**
Leu (g/d), *M* (*Q*_1_, *Q*_3_)	7.55 (6.40, 8.78)	7.63 (6.45, 8.84)	7.30 (6.16, 8.30)	*Z* = −3.06^b^	**0.002**
Val (g/d), *M* (*Q*_1_, *Q*_3_)	4.95 (4.17, 5.89)	5.00 (4.20, 5.95)	4.79 (4.06, 5.60)	*Z* = −3.16^b^	**0.002**

a χ^2^ test (*P* < 0.05).

b Mann–Whitney *U* test.

M, median; *Q*_1_: 1st quartile; *Q*_3_: 3rd quartile. Bold values indicate statistically significant results (*P* < 0.05).

**Figure 3:**
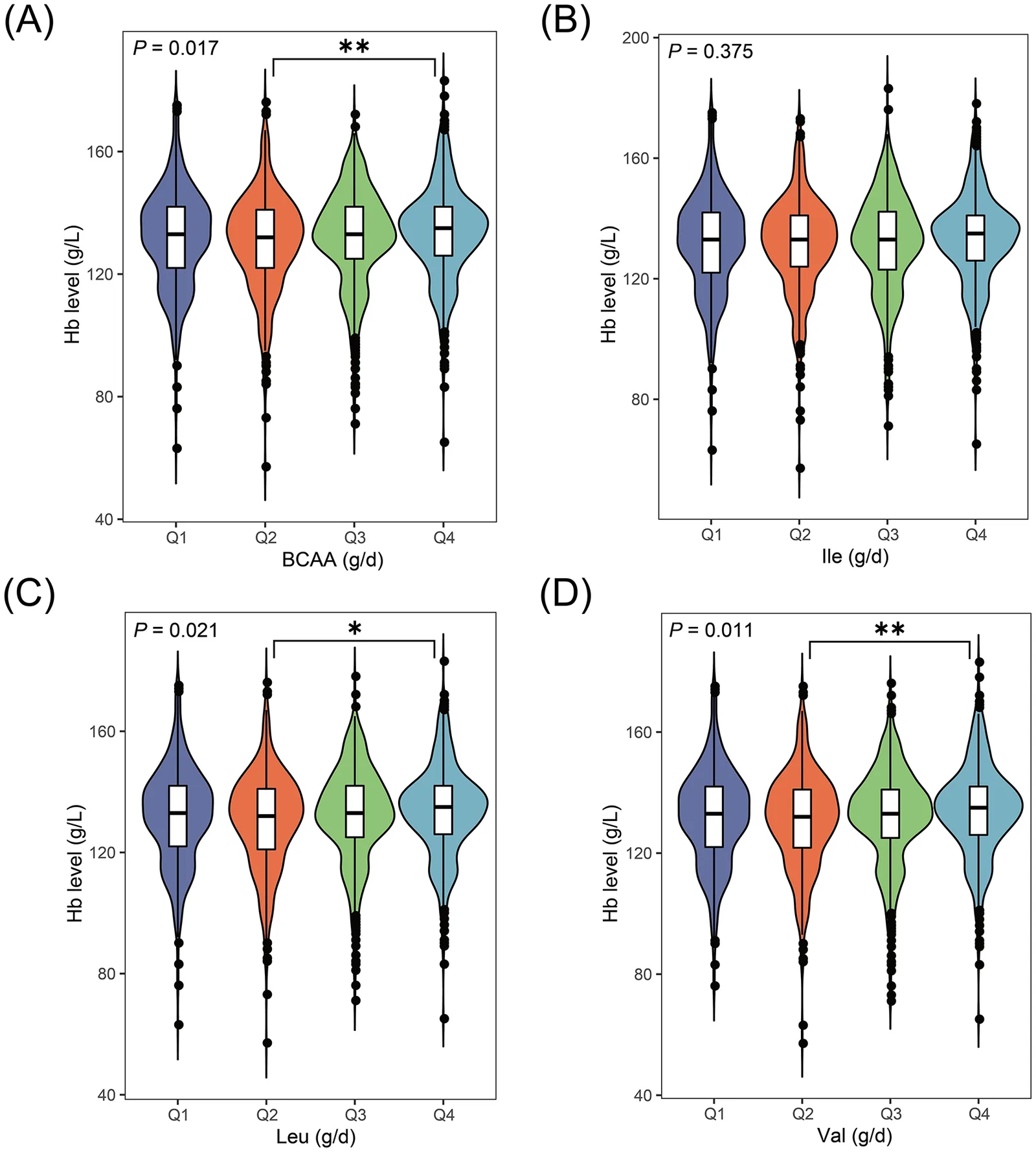
Violin plots of Hb levels stratified by quartiles of dietary branched-chain amino acid (BCAA) intake. **(A)** Total BCAA; **(B)** Ile; **(C)** Leu; **(D)** Val. *P < 0.05, **P < 0.01.

Supplementary biomarker analyses showed that the anaemia group had lower ferritin and higher sTfR concentrations (*P* < 0.001), whereas hsCRP did not differ between groups (*P* > 0.05). Albumin, serum total protein, zinc and vitamin A concentrations were also lower in the anaemia group (Supplementary Table S1).

Table 3 shows that higher intakes of total BCAA, Ile, Leu, and Val were consistently associated with lower odds of anaemia across the three conditional logistic regression models (*P* < 0.05). Model 1 was the crude model without adjusting for confounding factors; Model 2 adjusted for age, sex, and BMI; Model 3 adjusted for age, sex, BMI, physical activity, and energy intake. After full adjustment, the ORs for total BCAA, Ile, Leu, and Val were 0.95 (0.92–0.99), 0.82 (0.70–0.95), 0.91 (0.85–0.98), and 0.86 (0.78–0.95), respectively.

**Table 3:** Conditional logistic regression analysis of dietary branched-chain amino acid intake and anaemia.

**Variables**	Model 1		Model 2		Model 3	
	**OR (95% CI)**	***P***	**OR (95% CI)**	***P***	**OR (95% CI)**	***P***
BCAA	0.96 (0.92–0.99)	**0.006**	0.96 (0.92–0.99)	**0.007**	0.95 (0.92–0.99)	**0.006**
Ile	0.82 (0.70–0.96)	**0.011**	0.82 (0.70–0.96)	**0.012**	0.82 (0.70–0.95)	**0.011**
Leu	0.91 (0.85–0.98)	**0.009**	0.91 (0.85–0.98)	**0.010**	0.91 (0.85–0.98)	**0.009**
Val	0.86 (0.78–0.95)	**0.004**	0.86 (0.78–0.95)	**0.004**	0.86 (0.78–0.95)	**0.003**

Conditional logistic regression was used to estimate odds ratios (ORs) and 95% confidence intervals (CIs) (*P* < 0.05). Bold values indicate statistically significant results (*P* < 0.05).

Model 1: unadjusted.

Model 2: adjusted for age, sex, and BMI.

Model 3: adjusted for age, sex, BMI, physical activity, and energy intake.

### 3.3 Machine learning and sensitivity analyses

In exploratory machine learning analyses, LightGBM showed the highest descriptive AUC among the four models [AUC = 0.903 (0.886–0.919); Figure 4]. SHAP analysis was then used to rank variables in the LightGBM model, and the BCAA-related variables were ranked as Leu, Ile, and Val (Figure 5).

**Figure 4:**
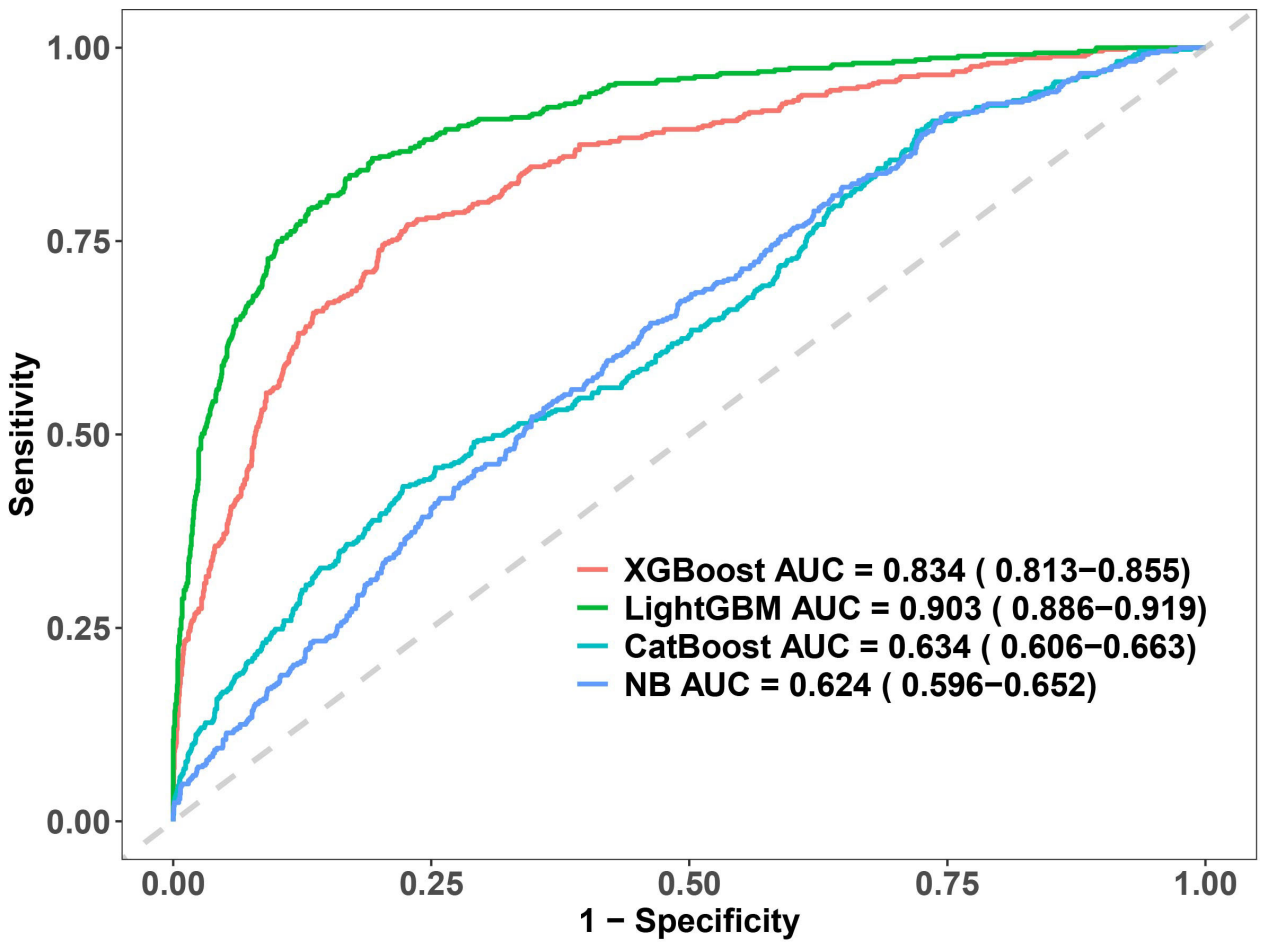
Receiver operating characteristic (ROC) curves for the four machine learning models.

**Figure 5:**
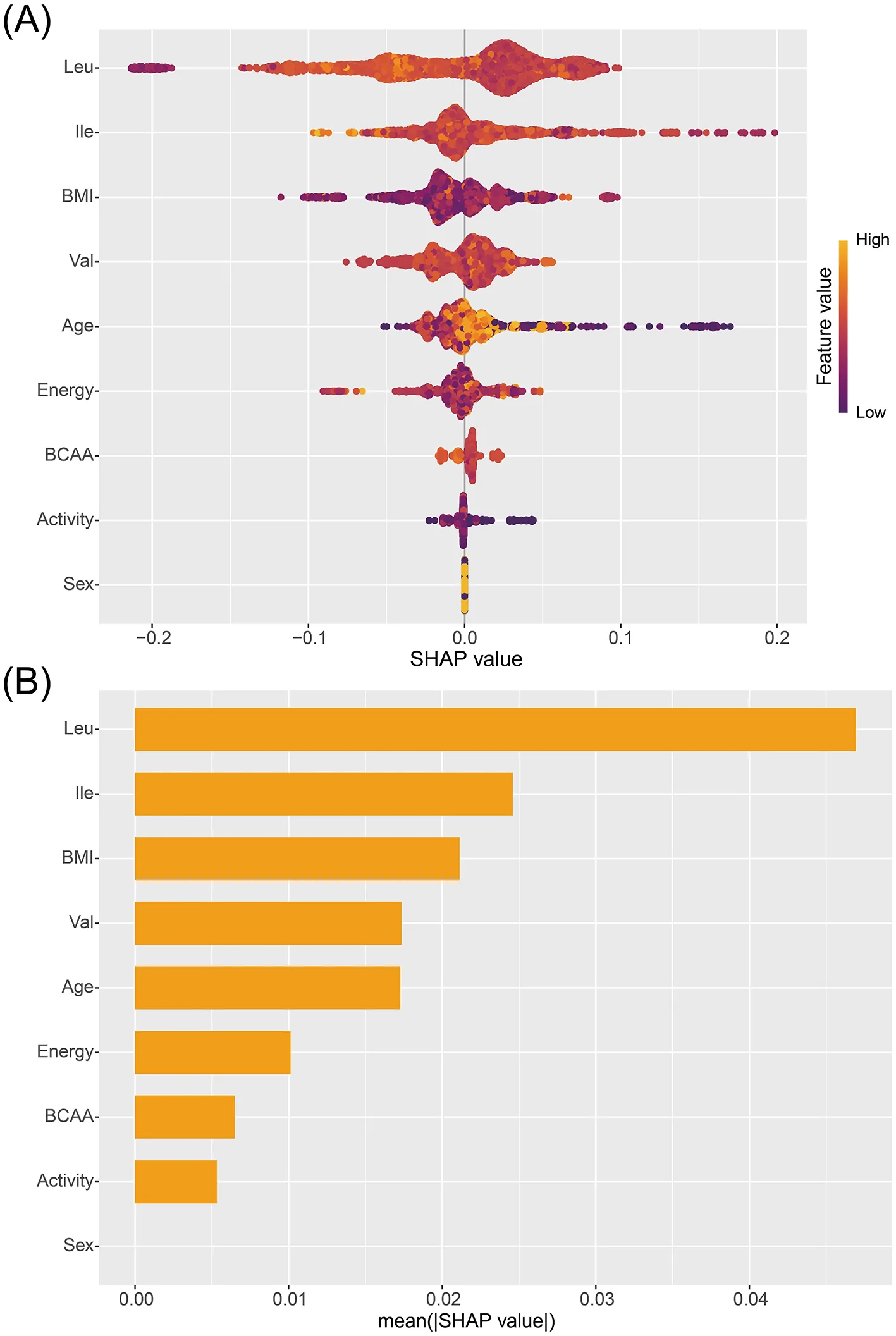
SHAP based interpretation of the LightGBM model. **(A)** SHAP summary plot showing the most important features and their impact on model output. Each point represents one participant; purple indicates lower feature values, and yellow indicates higher feature values. **(B)** Feature importance ranking based on SHAP analysis.

Conditional logistic regression showed inverse associations between total BCAA, Ile, Leu and Val intake and anaemia; stratified and RCS analyses were therefore performed to further examine these associations. In stratified analyses, statistically significant inverse associations were observed among children aged 6–10 years and among those with normal weight. However, likelihood ratio tests did not show statistically significant interactions between dietary BCAA intake and age, sex, BMI, physical activity or energy intake. Therefore, subgroup findings should be interpreted as exploratory patterns rather than evidence of effect modification (Figure 6). RCS results showed that, after adjusting for confounding factors, there was a significant nonlinear dose-response relationship between total BCAA, Leu, and Val intake and anaemia risk (*P* for nonlinear < 0.01), while Ile intake showed an approximately linear inverse association, with no evidence of nonlinearity (*P* for nonlinear > 0.05), although the overall spline test did not reach conventional statistical significance (*P* = 0.068; Figure 7).

**Figure 6:**
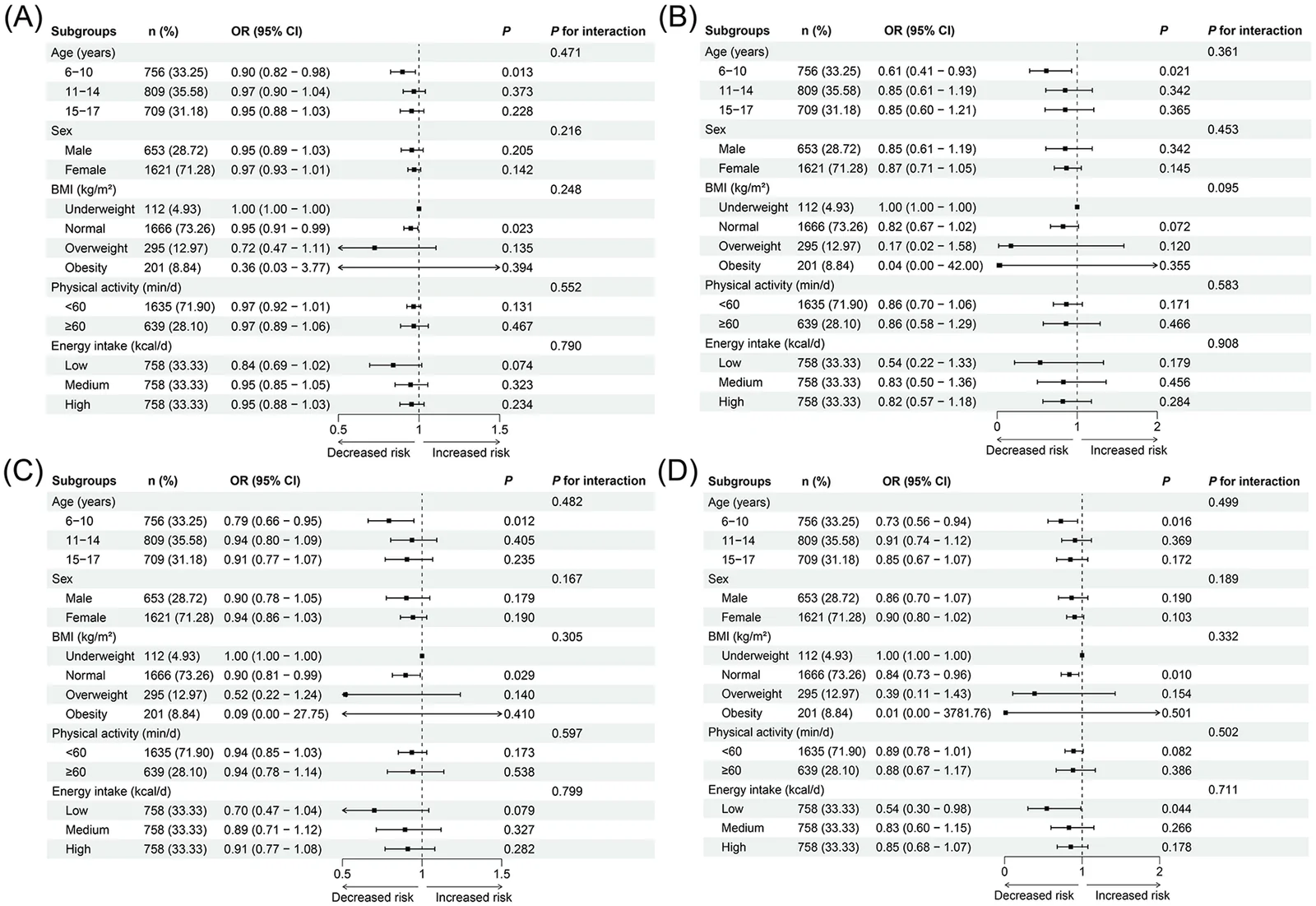
Forest plots of subgroup analyses of the association between dietary BCAA intake and the odds of anaemia. **(A)** Total BCAA; **(B)** Ile; **(C)** Leu; **(D)** Val. OR, odds ratio; CI, confidence interval.

**Figure 7:**
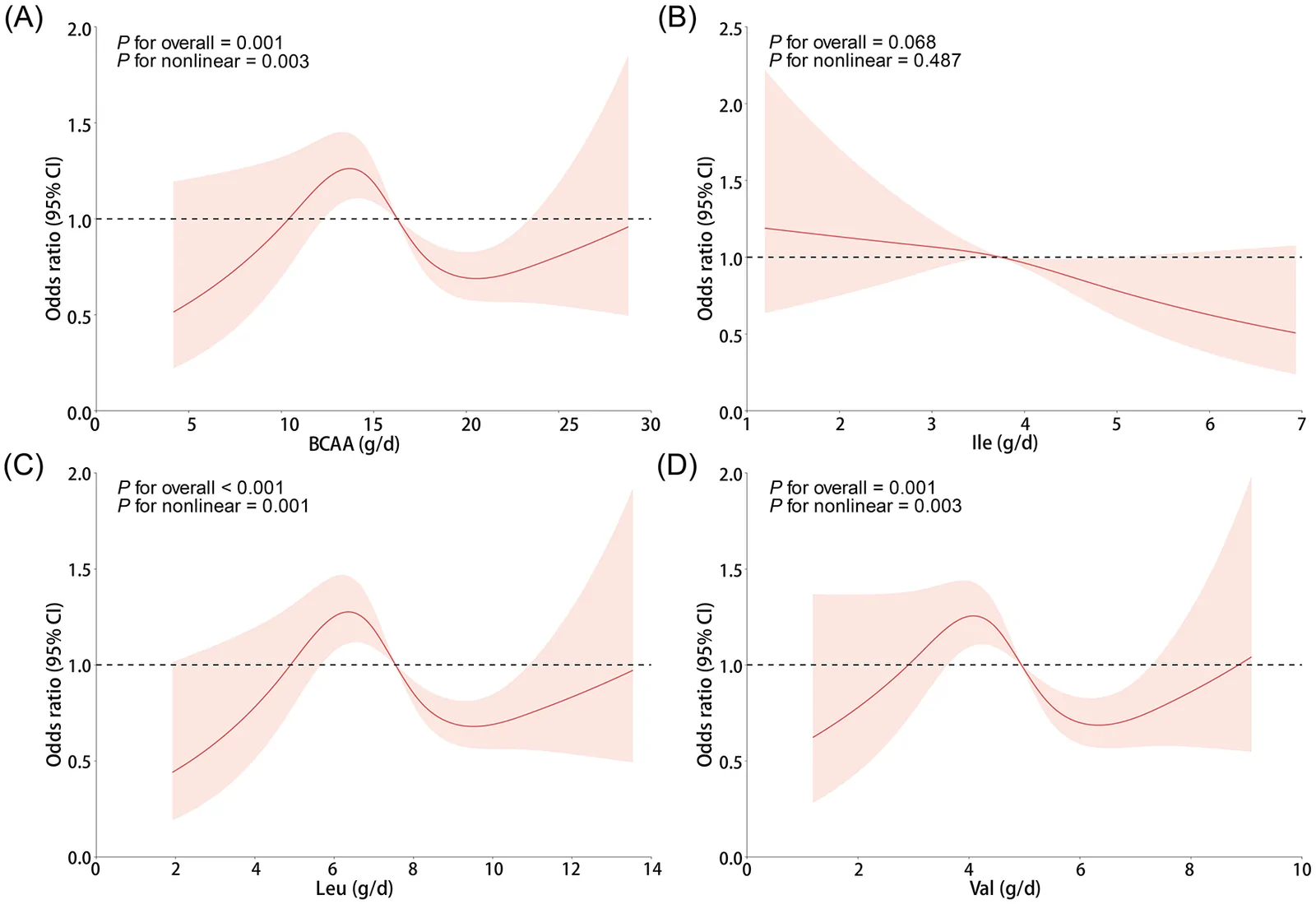
Restricted cubic spline (RCS) analyses of dietary BCAA intake and the odds of anaemia. **(A)** Total BCAA; **(B)** Ile; **(C)** Leu; **(D)** Val. CI, confidence interval.

## 4 Discussion

This study found that higher dietary BCAA intake was consistently associated with lower odds of Hb-defined anaemia in children and adolescents. After full adjustment, the ORs for Ile, Leu and Val were 0.82 (0.70–0.95), 0.91 (0.85–0.98) and 0.86 (0.78–0.95), respectively. In stratified analyses, inverse associations were observed among children aged 6–10 years and among those with normal weight, although formal interaction tests did not support clear effect modification. Our findings are consistent with previous research on the relationship between BCAAs and iron metabolism indicators such as Hb and ferritin (5), and also consistent with the evidence that “better dietary patterns” or “higher dietary diversity” correspond to lower odds or lower prevalence of anaemia in children and adolescents (8, 9, 29). However, given the observational design and potential residual confounding, these findings should be interpreted as associations rather than evidence of an independent or protective effect of BCAA intake.

The supplementary biomarker results further support the biological plausibility of the haemoglobin-defined outcome. Lower ferritin and higher sTfR in the anaemia group suggested an unfavourable iron status, while the absence of a group difference in hsCRP did not indicate a higher systemic inflammatory burden. These findings are compatible with poorer iron status in the anaemia group, but they do not establish iron-deficiency anaemia as the specific underlying diagnosis. However, the lower zinc and vitamin A levels also suggest that the association between BCAA intake and anaemia should be interpreted in the context of broader nutritional status rather than as a BCAA-specific effect alone. Higher BCAA intake may also partly reflect better overall diet quality, including greater consumption of animal-source foods and higher intake of iron, vitamin B12 and zinc. The comparable total dietary protein intake between groups suggests that the lower BCAA intake in the anaemia group was not simply explained by lower overall protein intake, although differences in protein sources and micronutrient density may still be relevant.

Compared with previous studies, this study extends nutritional epidemiological research on anaemia in children and adolescents from the “food or dietary pattern level” to the “single essential amino acid component level.” Previous studies have focused more on the relationship between dietary patterns, dietary diversity, iron deficiency or iron-rich foods and anaemia/iron deficiency in children and adolescents (8, 9, 30), while there are still few population studies that directly focus on the relationship between total dietary BCAA and its components and anaemia. At the same time, previous machine learning studies on children's amino acid intake have focused more on the outcome of inflammation or metabolic risk indicators rather than anaemia (10). In terms of methodology, machine learning was used primarily as an exploratory feature-ranking approach to identify potentially important amino-acid-related variables, while conditional logistic regression provided the main estimates of association and sensitivity analyses assessed their robustness. Thus, the machine learning results should be viewed as supportive rather than independent evidence of the observed associations.

The inverse association observed between dietary BCAA intake and anaemia risk may reflect the comprehensive role of BCAAs in erythroid maturation, haem-related metabolism, and overall dietary quality. From a biological perspective, erythrocyte development and Hb synthesis are processes highly dependent on the supply of metabolic substrates. Recent reviews and basic research have shown that amino acid metabolism plays a key role in erythroid maturation, haem synthesis, and stress-induced haematopoiesis (6, 31–33). In addition, total BCAA, Leu, and Val showed non-linear dose-response associations with the odds of anaemia, whereas Ile showed an approximately linear inverse association. These nonlinear patterns suggest that the associations may vary across the observed intake range; however, specific intake thresholds or plateau points could not be reliably established from the present data. Subgroup analysis showed that total BCAA and its components had significant inverse associations in the 6–10 year age group. This result suggests that young children are still in a growth and development stage and may have higher requirements for iron, protein-rich foods and other micronutrients. Conversely, nutritional deficiencies are more likely to be reflected in an increased risk of anaemia (7, 34); total BCAA, Leu, and Val showed significant inverse associations in the normal-weight group, possibly due to obesity-related chronic inflammation interfering with iron homeostasis and weakening the correlation between nutritional factors and Hb (35, 36). It should be noted that no significant interaction was observed between any confounding factors and total BCAA intake and its components in this study. This suggests that the differences between subgroups should be interpreted as trends in association strength rather than explicit effect modifications. Overall, the negative correlation between dietary BCAA intake and anaemia risk was relatively consistent across subgroups, indicating a degree of robustness in the results.

These findings may have implications for nutritional assessment of anaemia in children and adolescents. In addition to iron status, dietary assessment may consider protein-rich food sources and essential amino-acid composition, particularly in groups with less favourable haematological or micronutrient profiles (37, 38). This does not mean that BCAAs can replace iron supplementation or clinical treatment, but rather that BCAA-related dietary characteristics may serve as supplementary nutritional clues in anaemia-related assessment. Given the modest effect sizes, these associations should be interpreted as population-level nutritional signals rather than evidence of substantial clinical effects.

Several limitations should be acknowledged. First, anaemia was defined according to haemoglobin concentration rather than a complete aetiological diagnosis. Although ferritin, sTfR and hsCRP were available, other indicators such as mean corpuscular volume, serum iron, transferrin saturation, total iron-binding capacity, folate and vitamin B12 were not available; therefore, the underlying aetiologies of anaemia could not be definitively classified, and the observed association should not be interpreted as being specific to any particular anaemia subtype. Second, the case-control design precludes conclusions about temporality or causality, and residual confounding may remain because information on dietary iron, vitamin B12, folate, protein sources, socioeconomic status and menstrual status was not fully available for adjustment. Third, dietary intake was assessed using an FFQ, which may be affected by recall bias and measurement error, particularly for amino-acid intake (39). Finally, machine learning and SHAP analyses were used for exploratory feature ranking, but variable importance is not equivalent to causal effect and this study lacked external validation. Future prospective studies with more comprehensive haematological, micronutrient and dietary-source data are needed to confirm these findings.

## 5 Conclusions

In this matched case-control study of Chinese children and adolescents, higher dietary BCAA intake was associated with lower odds of Hb-defined anaemia, despite comparable total dietary protein intake between groups. Supplementary biomarker analyses suggested that the anaemia group had less favourable iron and micronutrient status, supporting the nutritional relevance of the observed association. These findings indicate that BCAA-related dietary characteristics may provide useful clues for anaemia-related nutritional assessment, although prospective studies are needed to clarify the underlying pathways.

## Data Availability

The data analyzed in this study is subject to the following licenses/restrictions: the dataset is not publicly available due to data ownership, participant privacy and ethical restrictions. Access requires permission from the Chinese Center for Disease Control and Prevention and approval by the relevant study team or corresponding author. Requests to access these datasets should be directed to Lianlong Yu, lianlong00a@163.com.
