# Supplementary material for: Association between dietary branched-chain amino acid intake and anaemia risk in children and adolescents: a matched case-control study
Source: Front Nutr. 2026 Sep 28;13:1911612. doi: 10.3389/fnut.2026.1911612 (PMC13656852; doi:10.3389/fnut.2026.1911612)
Supplement: Supplementary file 1 [file Table_1.docx]

**Supplementary Table S1.** Iron-status, inflammatory and nutritional biomarkers after propensity score matching.

| **Variables** | **Total (n = 2274)** | **Normal (n = 1819)** | **Anaemia (n = 455)** | **Statistic** | ***P*** |
| --- | --- | --- | --- | --- | --- |
|  |  |  |  |  |  |
| Ferritin (µg/L), M (Q₁, Q₃) | 51.00 (29.74, 78.05) | 53.10 (34.27, 80.31) | 37.51 (8.74, 67.38) | *Z*=-9.05 | **<0.001** |
| Soluble transferrin receptor (mg/L),  M (Q₁, Q₃) | 3.34 (2.80, 4.01) | 3.28 (2.77, 3.87) | 3.96 (3.00, 5.84) | *Z*=-10.61 | **<0.001** |
| High-sensitivity C-reactive protein (mg/L), M (Q₁, Q₃) | 0.26 (0.15, 0.64) | 0.26 (0.15, 0.64) | 0.26 (0.15, 0.66) | *Z*=-0.21 | 0.832 |
| Albumin (g/L), M (Q₁, Q₃) | 49.00 (47.20, 51.00) | 49.30 (47.60, 51.23) | 48.00 (46.00, 50.20) | *Z*=-8.28 | **<0.001** |
| Serum total protein (g/L),  M (Q₁, Q₃) | 76.00 (73.00, 79.20) | 76.20 (73.10, 79.40) | 75.00 (72.05, 78.60) | *Z*=-3.84 | **<0.001** |
| Zinc (mg/L), M (Q₁, Q₃),  n=2212 | 0.88 (0.79, 0.98) | 0.89 (0.80, 0.98) | 0.85 (0.77, 0.96) | *Z*=-3.33 | **<0.001** |
| Vitamin A (mg/L), M (Q₁, Q₃),  n=2213 | 0.38 (0.32, 0.44) | 0.39 (0.32, 0.45) | 0.35 (0.29, 0.41) | *Z*=-7.22 | **<0.001** |

*Z* values were obtained from the Mann–Whitney U test. (*P* < 0.05). *P* < 0.05 was considered statistically significant (in bold). M, median; Q_1_: 1st quartile; Q_3_: 3rd quartile
